# RNA virus-mediated gene editing for tomato trait breeding

**DOI:** 10.1093/hr/uhad279

**Published:** 2023-12-19

**Authors:** Mireia Uranga, Verónica Aragonés, Arcadio García, Sophie Mirabel, Silvia Gianoglio, Silvia Presa, Antonio Granell, Fabio Pasin, José-Antonio Daròs

**Affiliations:** Instituto de Biología Molecular y Celular de Plantas (IBMCP), Consejo Superior de Investigaciones Científicas-Universitat Politècnica de València (CSIC-UPV), Avenida de los Naranjos s/n, 46022 Valencia, Spain; Instituto de Biología Molecular y Celular de Plantas (IBMCP), Consejo Superior de Investigaciones Científicas-Universitat Politècnica de València (CSIC-UPV), Avenida de los Naranjos s/n, 46022 Valencia, Spain; Instituto de Biología Molecular y Celular de Plantas (IBMCP), Consejo Superior de Investigaciones Científicas-Universitat Politècnica de València (CSIC-UPV), Avenida de los Naranjos s/n, 46022 Valencia, Spain; Instituto de Biología Molecular y Celular de Plantas (IBMCP), Consejo Superior de Investigaciones Científicas-Universitat Politècnica de València (CSIC-UPV), Avenida de los Naranjos s/n, 46022 Valencia, Spain; Instituto de Biología Molecular y Celular de Plantas (IBMCP), Consejo Superior de Investigaciones Científicas-Universitat Politècnica de València (CSIC-UPV), Avenida de los Naranjos s/n, 46022 Valencia, Spain; Instituto de Biología Molecular y Celular de Plantas (IBMCP), Consejo Superior de Investigaciones Científicas-Universitat Politècnica de València (CSIC-UPV), Avenida de los Naranjos s/n, 46022 Valencia, Spain; Instituto de Biología Molecular y Celular de Plantas (IBMCP), Consejo Superior de Investigaciones Científicas-Universitat Politècnica de València (CSIC-UPV), Avenida de los Naranjos s/n, 46022 Valencia, Spain; Instituto de Biología Molecular y Celular de Plantas (IBMCP), Consejo Superior de Investigaciones Científicas-Universitat Politècnica de València (CSIC-UPV), Avenida de los Naranjos s/n, 46022 Valencia, Spain; Instituto de Biología Molecular y Celular de Plantas (IBMCP), Consejo Superior de Investigaciones Científicas-Universitat Politècnica de València (CSIC-UPV), Avenida de los Naranjos s/n, 46022 Valencia, Spain

## Abstract

Virus-induced genome editing (VIGE) leverages viral vectors to deliver CRISPR-Cas components into plants for robust and flexible trait engineering. We describe here a VIGE approach applying an RNA viral vector based on potato virus X (PVX) for genome editing of tomato, a mayor horticultural crop. Viral delivery of single-guide RNA into Cas9-expressing lines resulted in efficient somatic editing with indel frequencies up to 58%. By proof-of-concept VIGE of *PHYTOENE DESATURASE* (*PDS*) and plant regeneration from edited somatic tissue, we recovered loss-of-function *pds* mutant progeny displaying an albino phenotype. VIGE of *STAYGREEN 1* (*SGR1*), a gene involved in fruit color variation, generated *sgr1* mutant lines with recolored red-brown fruits and high chlorophyll levels. The obtained editing events were heritable, overall confirming the successful breeding of fruit color. Altogether, our VIGE approach offers great potential for accelerated functional genomics of tomato variation, as well as for precision breeding of novel tomato traits.

## HIGHLIGHTS

- Generation of a transgenic Cas9-expressing line of tomato cv. Micro-Tom
- Use of PVX for sgRNA delivery into Cas9-expressing Micro-Tom plants
- Heritable proof-of-concept VIGE of tomato *PHYTOENE DESATURASE* (*PDS*)
- Recovery of *green-flesh* fruits by VIGE of tomato *STAYGREEN 1* (*SGR1*)
- VIGE of Cas9-expressing lines of Pera, a cultivated traditional tomato

## Introduction

Tomato (*Solanum lycopersicum* L.) is a mayor horticultural crop grown worldwide (https://www.fao.org/faostat/) and a model plant for studying the genetics of fleshy fruit and domestication traits. To address the limited genetic diversity of modern tomatoes and facilitate the development of new varieties with enhanced traits, novel breeding approaches are needed [1, 2].

**Figure 1:**
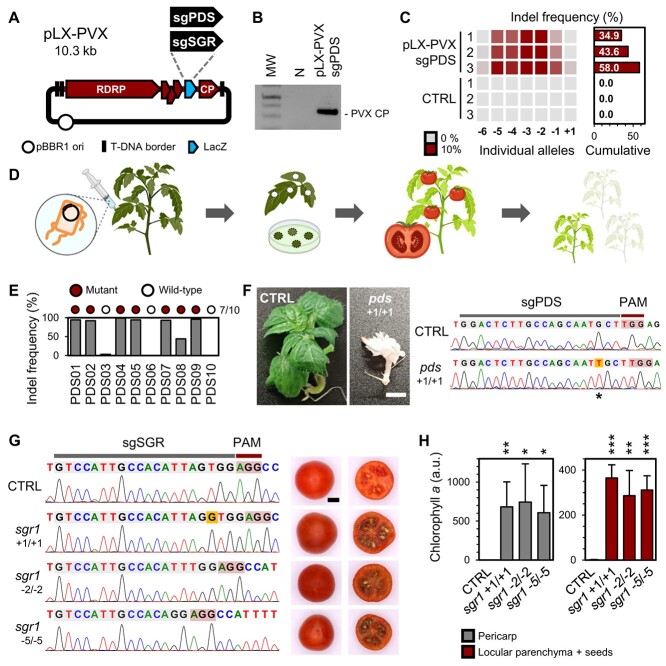
RNA virus/CRISPR-Cas9 editing in tomato. (**A**) Scheme of pLX-PVX, a mini T-DNA vector for agroinoculation of recombinant potato virus X (PVX) constructs for sgRNA delivery. The derivatives pLX-PVX::sgPDS and pLX-PVX::sgSGR include single-guide RNAs (sgRNAs) targeting the tomato *PHYTOENE DESATURASE* (*PDS*) and *STAYGREEN 1* (*SGR1*), respectively. (**B**) *Agrobacterium*-mediated inoculation of pLX-PVX in tomato. RT–PCR detection of a PVX genomic fragment (CP) in a Cas9-expressing tomato cv. Micro-Tom (MT-Cas9) plant agroinoculated with pLX-PVX::sgPDS; samples were collected from upper uninoculated leaves; MW, DNA size standards; N, negative control. (**C**) Virus-induced genome editing (VIGE) of *PDS* in tomato somatic cells. MT-Cas9 plants were agroinoculated with pLX-PVX::sgPDS; after 6 weeks, VIGE was assessed in upper uninoculated leaf samples by PCR of a *PDS* genomic fragment and Sanger trace deconvolution. Indel frequency percentages are shown; CTRL, uninoculated MT-Cas9 plants. (**D**) Experimental workflow for tomato trait breeding using the PVX/CRISPR-Cas9 system. sgRNAs are delivered into MT-Cas9 by pLX-PVX agroinoculation, whole plants are regenerated *in vitro* from upper uninoculated leaf tissue, and progeny is screened for mutant line identification. (**E**) VIGE of *PDS* in regenerated tomato plants. Indel frequency percentages and genotypic classification of 10 regenerated plants are shown. (**F**) Rescue of homozygous *PDS* mutant progeny. Phenotype and Sanger trace of the progeny line PDS02.3 with a homozygous loss-of-function *PDS* allele (*pds* +1/+1); CTRL, control condition. (**G**) Rescue of homozygous *SGR1* mutant progeny. Sanger traces and mature fruit phenotypes of progeny lines with homozygous loss-of-function *SGR1* alleles; CTRL, unedited MT-Cas9. (**H**) Chlorophyll quantification in fruits of *SGR1* mutants. Normalized fluorometric amounts of chlorophyll *a* in mature fruit samples are plotted (mean ± SD, *n* = 4); significance levels versus the control unedited MT-Cas9 (CTRL) as per Student’s *t*-test are shown; ^*^, *p* ≤ 0.05; ^**^, *p* ≤ 0.01; ^***^, *p* ≤ 0.001.

Owing to their specificity and versatility, clustered regularly interspaced short palindromic repeats (CRISPR)-CRISPR-associated protein (Cas) systems are widely used for efficient genome editing in numerous plant species, including tomato [3–6]. Current genome editing methods mainly use the *Streptococcus pyogenes* Cas9 nuclease and single-guide RNA (sgRNA) molecules that are delivered into plant cells through stable *Agrobacterium*-mediated transformation of constructs for the simultaneous Cas9 and sgRNA expression. Although powerful, these methods may require careful sgRNA design and combinatorial T-DNA construct assembly, selection of stable transformation events to recover transgenic plants, as well as complex breeding schemes to eventually obtain the desired mutant lines [7].

Virus-induced genome editing (VIGE) leverages viral vectors to transiently deliver CRISPR-Cas components into plants [8]. Compared to alternative methods, VIGE (i) allows efficient and fast genome editing for rapid prototyping of sgRNA designs, (ii) minimizes the plant genome integration of exogenous DNA if based on RNA viral vectors, (iii) allows recovery of virus-free edited progeny under optimized conditions [9]. VIGE has been successfully applied to model plants [10–14], but not-yet reported for horticultural trait breeding of tomato. Given the VIGE great potential for advanced breeding strategies of this important crop, we developed and describe here a VIGE approach based on an RNA viral vector for tomato genome engineering. We show that the obtained modifications at target genomic loci are heritable, and our VIGE approach can be used for horticultural trait breeding of tomato.

## Results

### Proof-of-concept VIGE of tomato *PHYTOENE DESATURASE* (*PDS*)

We previously reported efficient multiplex, heritable editing in *Nicotiana benthamiana* by infection of a transgenic line constitutively expressing Cas9 with a potato virus X (PVX; genus *Potexvirus*) vector for sgRNA expression in plant cells [12, 15]. PVX infects a wide range of Solanaceae species. To translate our reported VIGE approach to tomato, we first generated a transgenic Cas9-expressing line of the cultivar Micro-Tom (MT-Cas9) by *Agrobacterium*-mediated stable transformation. T_0_ plants were selected on kanamycin-supplemented medium and by visual tracking of the expression of a reporter gene (DsRed); transgene integration was confirmed by PCR amplification of a T-DNA fragment from plant genomic DNA. We next engineered pLX-PVX, an enhanced PVX vector based on a mini binary vector of the pLX series [16] for autonomous replication in *Escherichia coli* and *Agrobacterium*. pLX-PVX allows *Agrobacterium*-mediated inoculation (agroinoculation) of PVX and virus-mediated sgRNA expression in plants (Figure 1A).

We reasoned that PVX-mediated sgRNA expression in MT-Cas9 would result in editing events of the tomato genome. For proof-of-concept VIGE assays, we assembled pLX-PVX::sgPDS for expression of a sgRNA targeting *PHYTOENE DESATURASE* (*PDS*) (Figure S1A; Table S1 of Supplementary Data), whose loss-of-function mutants have a reported photobleaching phenotype [17]. Cotyledons of MT-Cas9 seedlings were agroinoculated with pLX-PVX::sgPDS, and samples were collected from upper uninoculated leaves after 6 weeks. PVX infectivity in inoculated plants was confirmed by RT–PCR detection of a viral fragment (Figure 1B). Although no photobleaching could be observed (Figure S1B), PCR amplification of a plant genomic fragment spanning the sgRNA target site followed by Sanger trace deconvolution revealed the presence of multiple *PDS* alleles with insertion–deletion (indel) mutations and cumulative indel frequencies of 34.9–58.0% (Figure 1C). These results confirm that RNA virus-mediated sgRNA delivery can produce somatic mutations in a tomato Cas9-expressing line.

Given the high editing efficiency detected in somatic cells, we conceived an experimental workflow for heritable trait breeding using the PVX/CRISPR-Cas9 system in tomato (Figure 1D). At 21 dpi, we collected upper uninoculated leaves of infected plants, detected *PDS* indel frequencies of 19.7–46.9% (*¯* = 28.5; Figure S1C), and regenerated whole plants by tissue culture (Figure 1D). Genotyping of randomly picked green plants indicated that 70% (7/10) of them had *PDS* mutant alleles (Figure 1E). Among these, the line PDS02 had a 1-bp insertion allele (+1) and a 3-bp deletion allele (–3) in *PDS* (Table S2). To confirm the mutation heritability, fruits were collected from the self-pollinated PDS02 line and progeny analyzed. The recovered lines PDS02.1 and PDS02.2 were bi-allelic (+1/–3) or homozygous (–3) mutants showing the green, wildtype phenotype (Figure S1D; Table S2). Remarkably, the progeny line PDS02.3 showed a photobleaching phenotype and the homozygous +1 allele (*pds* +1/+1; Figure 1F; Table S2), which caused the *PDS* complete inactivation. These findings demonstrate that our VIGE approach yields, through tissue culture regeneration, heritable editing events and can be used to recover loss-of-function mutant progeny.

### VIGE of tomato *STAYGREEN 1* (*SGR1*) yields fruits with the *green-flesh* phenotype

Fruit coloration is an important trait for fresh market tomatoes, and it is influenced by multiple genes that control pigment accumulation in the pericarp and peel [7, 18, 19]. Heirloom accessions show a wide range of fruit color variation; among others, Purple Calabash, Cherokee Chocolate, Black Krim, and Black Prince are mutants of *STAYGREEN 1* (*SGR1*) [20]. *SGR1* inactivation inhibits degradation of chlorophyll *a*; simultaneous chlorophyll and lycopene accumulation during ripening produces a *green-flesh* phenotype characterized by red-brown fruits [7, 19].

Supported by the *PDS* editing results, we wondered if our VIGE approach could be used for *SGR1* editing and breeding of fruit color. We thus assembled pLX-PVX::sgSGR to target *SGR1* (Figures 1A,S2A; Table S1), and agroinoculated it into MT-Cas9 seedlings. At 21 dpi, Sanger trace deconvolution analysis revealed an average *SGR1* indel frequency of 21.4% in upper uninoculated leaves (Figure S2B), which were then used for in-vitro regeneration of whole plants. 60% (9/15) of them had *SGR1* mutant alleles (Figure S2C). Subsequent fruit collection and progeny analysis allowed identifying homozygous lines with a single mutant allele of *SGR1* each (*sgr1* +1/+1, *sgr1* –2/–2, and *sgr1* –5/–5), and whose fruits displayed the *green-flesh* phenotype (Figure 1G); no PVX could be detected in these lines (Figure S3). Fluorometric and absorbance-based quantification revealed a significant increase of chlorophyll *a* amount in the pericarp and locular parenchyma plus seed samples of *sgr1* mutant lines compared to the control, unedited MT-Cas9 (Figures 1H,S4A). Results of the two complementary methods were highly consistent (*r* = 0.967, *p* < 0.0001; Figure S4B), overall confirming the *SGR1* inactivation. Altogether, our VIGE results prove the successful recovery of virus-free, *SGR1* loss-of-function mutants and thus horticultural trait breeding of tomato.

### PVX-based VIGE of traditional tomatoes

We asked if our PVX-based VIGE approach could be applied to cultivated tomatoes. Traditional varieties show high stability of agro-morphological traits [2] and include Muchamiel and Pera, two cultivars popular in Spain. PVX infectivity was confirmed in Muchamiel and a derived line with introgressed *Tm-2^2^*, *Sw-5*, and *Ty-1* loci conferring resistance to tobamoviruses, tospoviruses, and geminiviruses, respectively (Figure S5). We then generated transgenic Cas9-expressing lines of Pera (Pera-Cas9; Figure S6A) that were agroinoculated with pLX-PVX vectors for sgRNA expression. Analyses of upper uninoculated leaves confirmed the PVX infectivity (Figure S6B-C), and revealed *PDS* and *SGR1* indel frequencies of 26–39% (*¯* = 32.8%) and 30–57% (*¯* = 44.9%), respectively (Figure S6D), overall, supporting the portability of our VIGE approach to cultivated tomato varieties.

## Discussion

We reported here (i) the generation of a transgenic Cas9-expressing line of tomato cv. Micro-Tom (MT-Cas9); (ii) use of pLX-PVX, a T-DNA plasmid with an enhanced RNA viral vector, for sgRNA delivery into MT-Cas9 plants; (iii) heritable, proof-of-concept VIGE of *PHYTOENE DESATURASE* and recovery of albino progeny; (iv) recovery of recolored *green-flesh* fruits by VIGE of *STAYGREEN 1*; and (v) generation of Cas9-expressing lines of a traditional cultivar and the successful editing of two genomic loci. Altogether, the results indicate that the presented VIGE approach can be readily applied for breeding of tomato traits with horticultural interest.

Simultaneous stable transformation of Cas nucleases and sgRNAs requires months until the DNA construct functionality is confirmed. Once edited lines are obtained, the continuous expression of sgRNA(s) and the selection marker used can limit subsequent transformation events and breeding for multiple traits. However, our Cas9-expressing lines with uncoupled sgRNA delivery would enable rapid prototyping of sgRNA designs within weeks, as well as unconstrained trait stacking through multiple VIGE rounds.

Zhenghe Li and co-workers recently reported a tospovirus vector system for viral delivery of Cas nucleases and their corresponding CRISPR guide RNAs, which enabled VIGE in a variety of non-transgenic crops [21]. Our potexvirus-based VIGE approach presents a valuable breeding resource for modern varieties with resistances that constrain the applicability of systems based on tospoviruses, tobamoviruses, and geminiviruses.

In sum, our work reinforces VIGE as a flexible and robust technology for accelerating functional genomics of tomato variation, as well as for precision breeding of novel traits to tackle global food supply [22–24] and health problems [25].

## Materials and methods

### Plasmid construction

The binary vector GB2234 for plant expression of a codon-optimized *S. pyogenes* Cas9 (hCas9) sequence under the control of the cauliflower mosaic virus (CaMV) 35S promoter and *Agrobacterium tumefaciens* nopaline synthase terminator (P_35S_:hCas9:T_nos_) was reported [12, 26]. GB2234 includes a kanamycin resistance gene (*npt*II) and a red fluorescent protein gene (DsRed) for plant selection.

pLX-PVX was used for recombinant viral vector assembly. Potato virus X (PVX) genomic cDNA were amplified from pPVX [12], a pgR107-derivative based on pGreen0000 which lacks autonomous replication in *Agrobacterium* [27]. The obtained viral sequence was inserted into pLX-B2 (GenBank: KY825137; Addgene: 160636), a mini T-DNA binary vector of the pLX series [16] for autonomous replication in *E. coli* and *Agrobacterium*. The T-DNA of pLX-PVX includes a full-length PVX cDNA flanked by the CaMV 35S promoter and terminator, single-guide RNA (sgRNA) expression is driven by the PVX coat protein (CP) promoter, whereas a PVX CP truncated version [28] is expressed from a heterologous promoter derived from bamboo mosaic virus. pLX-PVX includes the *LacZ* alpha peptide reporter for white-blue screen of recombinant vectors.

The sgRNA sequences of sgPDS and sgSGR, used to, respectively, target the tomato *PHYTOENE DESATURASE* (*PDS*; Solyc03g123760.2.1) and *STAYGREEN 1* (*SGR1*; Solyc08g080090.2), are shown in Table S1. Recombinant pLX-PVX derivatives for sgRNA expression were assembled using standard molecular biology techniques [15]. Briefly, fragments, including sgPDS and sgSGR fused to a truncated *Arabidopsis thaliana FLOWERING LOCUS T* sequence [10], were obtained by PCR reactions including high-fidelity Phusion DNA polymerase (Thermo Fisher Scientific), pPVX::sgXT2B-tFT as the DNA template [12], and the primer pairs D4747/D3898 and D4748/D3898, respectively (Table S3). Gel-purified fragments were inserted into MluI-linearized pLX-PVX by Gibson assembly reactions with the NEBuilder HiFi DNA assembly master mix (New England Biolabs). Recombinant plasmids were purified from white *E. coli* colonies selected on agar medium plates supplemented with kanamycin and 5-bromo-4-chloro-3-indolyl β-d-galactopyranoside (X-Gal). The obtained viral vectors pLX-PVX::sgPDS and pLX-PVX::sgSGR were verified by Sanger sequencing.

### Plant materials

The tomato cultivar ‘Micro-Tom’ and the Spanish traditional accessions ‘De la Pera 21’ (Pera) and ‘Muchamiel 18’ (M18) were reported, as well as UMH 1200, an M18-derived line with the introgressed *Tm-2^2^*, *Sw-5*, and *Ty-1* loci [29].

Tomato stable transformation was done using GB2234 as described [19]. Transformants of Micro-Tom (MT-Cas9) and Pera (Pera-Cas9) were identified by kanamycin selection and monitoring of the DsRed reporter expression under an epifluorescence stereoscope (Leica DMS1000). Genomic DNA was purified from plant samples using silica gel columns (Zymo Research), and stable transgene integration was confirmed by PCR amplification of a 792-bp T-DNA fragment using the primer pair D3663/D3664 (Table S3). Plants were grown in greenhouse chambers at ~24°C under a 16-hour day/8-hour night photoperiod.

### Viral vector agroinoculation and analysis of virus infectivity

*A. tumefaciens* C58C1 cells including a disarmed pTi were electroporated with viral vector plasmids; colonies were selected on plates supplemented with rifampicin and kanamycin. Suspensions of the transformed bacteria were prepared as described [15] and used to inoculate fully expanded cotyledons from 10-day-old seedlings.

Total RNA was purified from upper uninoculated leaf samples using silica gel columns as described [15]. cDNA was synthesized using RevertAid reverse transcriptase (Thermo Fisher Scientific) and the primer D2409 (Table S3); the PVX CP sequence was amplified in PCR reactions including the obtained cDNA, Tth DNA polymerase (Biotools) and the primer pair D2410/D3436 (Table S3). RT-PCR products were analyzed by electrophoresis in 1% agarose gels in TAE buffer (40 mM Tris, 20 mM sodium acetate, and 1 mM EDTA, pH 7.2), and visualized by ethidium bromide staining.

### *In vitro* regeneration of tomato plants

At 21 days post inoculation (dpi), systemic leaves showing symptoms of viral infection were detached, surface-sterilized by submersion in sterilization solution (10% bleach, 0.02% Nonidet P-40) for 10 min, and then rinsed three times in sterile water. One-cm leaf slices were obtained and plated on to callus induction medium including 1× Murashige & Skoog salts (MS) with vitamins, 0.5 g/L 2-(N-Morpholino)ethanesulfonic acid hydrate (MES), 20 g/L glucose, 10 g/L phytoagar, 0.1 mg/L 3-indolyl acetic acid (IAA), 0.75 mg/L trans-zeatin, pH 5.7. Leaf slices were transferred to fresh plates every 2–3 weeks until shoots emerged. The shoots were then transferred to elongation medium (1× MS with vitamins, 0.5 g/L MES, 20 g/L glucose, 10 g/L phytoagar, 0.1 mg/L trans-zeatin, pH 5.7) for 1–2 weeks until they reach a length of 1–2 cm. Subsequently, shoots were cut and transferred to rooting medium (0.5× MS with vitamins, 0.5 g/L MES, 20 g/L glucose, 10 g/L phytoagar, 0.1 mg/L IAA, pH 5.7). Rooted plantlets (>5 cm) were transferred to soil and covered with plastic cups to keep moisture in. After acclimation to soil conditions, plastic cups were removed for adequate plant growth. Fruits were harvested from each plant for progeny analysis.

### Genome editing analysis

Genomic DNA was purified from leaf samples using silica gel columns [15]. PCR using high-fidelity Phusion DNA polymerase, and the primer pairs D4762/D4763 and D4766/D4767 (Table S3) were respectively used to amplify tomato genomic fragments spanning the sgPDS and sgSGR target sites. PCR products were separated by agarose gel electrophoresis, purified, and subjected to Sanger sequencing. Genome editing efficiency was quantified by Sanger trace deconvolution [15] using TIDE (http://shinyapps.datacurators.nl/tide-batch/) or ICE (https://ice.synthego.com/).

### Chlorophyll quantification

Samples were collected from red mature tomato fruits. Aliquots of pericarp tissue (±100 mg) and seeds plus the surrounding locular parenchyma (±50 mg) were, respectively, mixed with 10 or 20 volumes (~1 mL) of aqueous 80% (v/v) acetone precooled to −20°C, homogenized using a ball mill (Star-Beater, VWR) for 5 minutes at 30 s^−1^, and mixed (1 h, 250 rpm) at 4°C in the dark. Samples were centrifuged at 14,000×*g* (5 minutes) to remove cell debris; supernatants were collected and kept in the dark until analysis.

Fluorometric quantification of chlorophyll *a* (chl *a*) was done in a monochromator-based plate reader (Infinite M Plex, Tecan Group) as reported [30, 31], with minor modifications. Briefly, the above-prepared supernatant samples were mixed 1:1 with aqueous 80% (v/v) acetone or aqueous 80% (v/v) acetone, 0.02 N HCl, to obtain native (*N*) and acidified (*A*) extracts, respectively. Samples were aliquoted to a black 96-well plate (Nunc), and top reading measurements recorded using λ_Ex_ 436 nm and λ_Em_ 680 nm; the instrument gain value was manually adjusted to avoid signal saturation. Fluorescence intensity of *N* and *A* extracts was measured to obtain F*_N_* and F*_A_*, respectively; chl *a* amount was calculated as F*_N_* – F*_A_* with background values subtracted.

Spectrophotometric quantification of chl *a* was done as reported [32]. Briefly, supernatant absorbance was measured at 646 nm (A_646_) and 663 nm (A_663_) against an aqueous 80% (v/v) acetone blank in a plate reader (Infinite M Plex, Tecan Group), and chl *a* quantified using the equation:

\begin{align*} \text{Chl}\ a=12.21^\ast\text{A}_{663}-2.81^{\ast} \text{A}_{646} \end{align*}

### Statistics

Student's *t* test was used for two-group comparisons; significance levels of *p* values are indicated in the figures. Pearson correlation coefficient was calculated to estimate the linear relationship between the two variables.

## Acknowledgements

This work was supported by grant PID2020-114691RB-I00 from Ministerio de Ciencia e Innovación (Spain) through the Agencia Estatal de Investigación (Spain), and by the program PROMETEO CIPROM/2022/21 of Generalitat Valenciana (Spain). An.G. received funding from Horizon 2020 with HARNESSTOM contract 101000716 (European Commission). F.P. is supported by the ‘Juan de la Cierva Incorporación’ contract IJC2019-039970-I and Ar.G. by FPU20/05477 from Ministerio de Ciencia e Innovación (Spain); M.U. by the Marie Skłodowska-Curie Actions (HORIZON-MSCA-2022-PF-01-101110621) from the European Commision; S.G. by CIAPOS/2021/316 from Generalitat Valenciana (Spain) and European Social Fund. We thank C. Gómez-Mena (IBMCP, Spain) for discussion, and S. García-Martínez and J.J. Ruiz (Universidad Miguel Hernández, Spain) for materials.

## Author contributions

All authors participated in the work conception and design. M.U., V.A., Ar.G., S.M., S.G. and S.P. performed the experiments. All authors participated in the result and data analyses. M.U., F.P. and J.-A.D. wrote the manuscript with input from the rest of the authors.

## Data availability

Supplementary data accompany this article. The pLX-PVX vector is available at Addgene with catalog number 213040 (https://www.addgene.org/213040/). The sequence information of tomato *PDS* (Solyc03g123760) and *SGR1* (Solyc08g080090) are available at Solanaceae Genomics Network (https://solgenomics.net/); GB2234 sequence is available at the GoldenBraid database (https://gbcloning.upv.es/feature/GB_UA_2578/). Further information and requests for resources should be directed to and will be fulfilled by the corresponding authors.

## Conflict of interest statement

The authors declare no conflict of interest.

## Supplementary Data

Supplementary data is available at Horticulture Research online.

## Supplementary Material

Web_Material_uhad279
