# Supplementary material for: RNA virus-mediated gene editing for tomato trait breeding
Source: Hortic Res. 2023 Dec 19;11(1):uhad279. doi: 10.1093/hr/uhad279 (PMC11184526; doi:10.1093/hr/uhad279)
Supplement: Web_Material_uhad279 [file web_material_uhad279.pdf]

SUPPLEMENTARY DATA

RNA virus-mediated gene editing for tomato trait breeding

Mireia Uranga[1, 2, †], Verónica Aragonés[1, †], Arcadio García[1, †], Sophie Mirabel[1], Silvia Gianoglio[1, †], Silvia Presa[1], Antonio Granell[1, †], Fabio Pasin[1, †, *], José-Antonio Daròs[1, †, *]

[1] Instituto de Biología Molecular y Celular de Plantas (IBMCP), Consejo Superior de Investigaciones Científicas – Universitat Politècnica de València (CSIC-UPV), 46022 Valencia, Spain; Emails: M.U., miurrui@alumni.upv.es; V.A., varagoni@ibmcp.upv.es; Ar.G., arcadiogarcia@ibmcp.upv.es; S.M., smirabel@ibmcp.upv.es; S.G., sgianog@ibmcp.upv.es; S.P., silviapr@upvnet.upv.es; An.G., agranell@ibmcp.upv.es; F.P., f.pasin@csic.es; J.-A.D., jadaros@ibmcp.upv.es

[2] Present address: Laboratory for Plant Genetics and Crop Improvement, Division of Crop Biotechnics, Department of Biosystems, KU Leuven, Heverlee, Belgium; KU Leuven Plant Institute (LPI), KU Leuven, Heverlee, Belgium.

[†] **ORCID:** M.U., 0000-0001-6343-4031; V.A., 0000-0002-4695-7890; Ar.G., 0009-0001-6425-2317; S.G., 0000-0003-3210-2020; An.G., 0000-0003-4266-9581; F.P., 0000-0002-9620-4301; J.-A.D., 0000-0002-6535-2889

*** Correspondence:**

Fabio Pasin, IBMCP (CSIC-UPV), Avenida de los Naranjos, s/n, 46022 Valencia, Spain; Tel.: +34 963877893; Fax. +34 963877859; Email: f.pasin@csic.es

José-Antonio Daròs, IBMCP (CSIC-UPV), Avenida de los Naranjos, s/n, 46022 Valencia, Spain; Tel.: +34 963877893; Fax. +34 963877859; Email: jadaros@ibmcp.upv.es

TABLE OF CONTENTS

SUPPLEMENTARY TABLES

Table S1. Single-guide RNAs used in the study

Table S2. Heritability of *PHYTOENE DESATURASE* (*PDS*) mutations

Table S3. Primers used in the study

SUPPLEMENTARY FIGURES

Figure S1. Virus-induced genome editing (VIGE) of tomato *PHYTOENE DESATURASE* (*PDS*)

Figure S2. VIGE of tomato *STAYGREEN 1* (*SGR1*)

Figure S3. PVX detection in *SGR1* mutant lines

Figure S4. Chlorophyll spectrophotometric quantification in fruits of *SGR1* mutant lines

Figure S5. PVX infection of cultivated tomato varieties

Figure S6. Somatic gene editing of Pera, a cultivated traditional tomato

SUPPLEMENTARY REFERENCES

SUPPLEMENTARY TABLES

Table S1. Single-guide RNAs used in the study

sgRNA	Target gene	Target gene accession number	Sequence	Ref.
sgPDS	PHYTOENE DESATURASE	Solyc03g123760	GGACTCTTGCCAGCAATGCT	[1]
sgSGR	STAYGREEN 1	Solyc08g080090	GTCCATTGCCACATTAGTGG	[2]

Table S2. Heritability of *PHYTOENE DESATURASE* (*PDS*) mutations

Line[a]	Allele	Allele sequence[b]	Allele frequency[c]
Control	wild-type	GGACTCTTGCCAGCAATGCTTGG	—
PDS02	+1	GGACTCTTGCCAGCAATTGCTTGG	47%
	-3	GGACTCTTGCCAGC---GCTTGG	44%
PDS02.1	+1	GGACTCTTGCCAGCAATTGCTTGG	48%
	-3	GGACTCTTGCCAGC---GCTTGG	46%
PDS02.2	-3	GGACTCTTGCCAGC---GCTTGG	74%
PDS02.3	+1	GGACTCTTGCCAGCAATTGCTTGG	97%

[a] The *in vitro* regenerated line PDS02 and its progeny lines PDS02.1, PDS02.2, and PDS02.3 are shown
[b] sgPDS target sequence with indels in mutant alleles shown in red; the protospacer adjacent motif (PAM) is underlined
[c] Frequencies by Sanger trace deconvolution

Table S3. Primers used in the study

Primer	Primer sequence (5'→3')	Purpose
D4747	GAGGTCAGCACCAGCTAGCAGGACTCTTGCCAGCAATGCTGTTTTAGAGC	sgRNA cloning
D4748	GAGGTCAGCACCAGCTAGCAGTCCATTGCCACATTAGTGGGTTTTAGAGC	sgRNA cloning
D3898	GGGAAACTTAACAAACCCTATTGGCCATAAGTAACCTTTAG	sgRNA cloning
D2409	ATTTATATTATTCATACAATCAAACC	PVX detection
D3436	ATGTCAGGCCTGTTCACTATCC	PVX detection
D2410	TGGTGGTGGTAGAGTGACAAC	PVX detection
D3663	ATGATTGAACAAGATGGATTGC	Plant genotyping
D3664	GAAGAACTCGTCAAGAAGGCGA	Plant genotyping
D4762	TCTGTATTTTGTCTAGCTTCTCCT	Plant genotyping
D4763	TGCACTGCATTGAAAGTTCGTC	Plant genotyping
D4766	TCCGTGCCGTCTATTGTTCT	Plant genotyping
D4767	ACCCAAACTAAAGCTTCTTGTAACT	Plant genotyping

SUPPLEMENTARY FIGURES

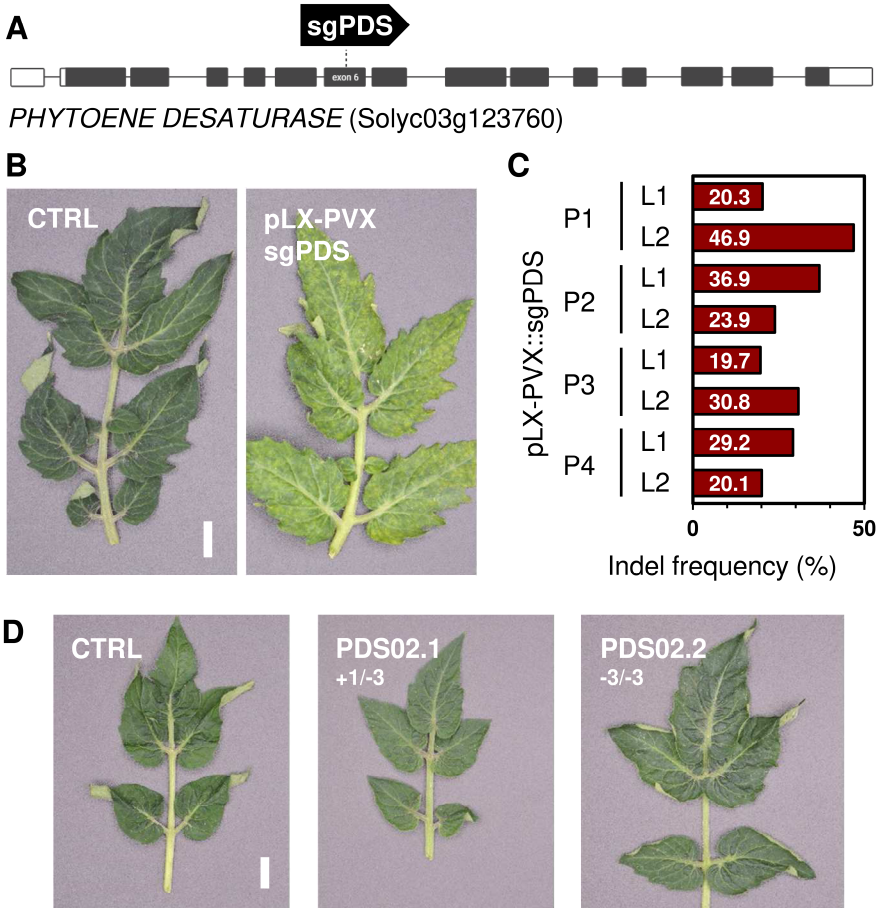

Figure S1. Virus-induced genome editing (VIGE) of tomato *PHYTOENE DESATURASE* (*PDS*). (**A**) Genetic structure of the tomato *PDS* (Solyc03g123760) and the pLX-PVX::sgPDS target region in exon 6. (**B**) Phenotype of a Cas9-expressing tomato cv. Micro-Tom (MT-Cas9) plant after 6 weeks of agroinoculation with pLX-PVX::sgPDS; CTRL, untreated plant; scale = 1 cm. (**C**) VIGE of *PDS* in tomato somatic cells. MT-Cas9 plants were agroinoculated with pLX-PVX::sgPDS; after 21 days, VIGE was assessed in upper uninoculated leaf samples by PCR of a *PDS* genomic fragment and Sanger trace deconvolution. Indel frequency percentages are shown for four plants (P1 – P4) and two leaves each (L1 – L2). (**D**) Pictures show the phenotypes of PDS02.1 and PDS02.2, progeny lines of a single plant (PDS02) regenerated from MT-Cas9 agroinoculated with pLX-PVX::sgPDS; see Table S2 for genotyping results.

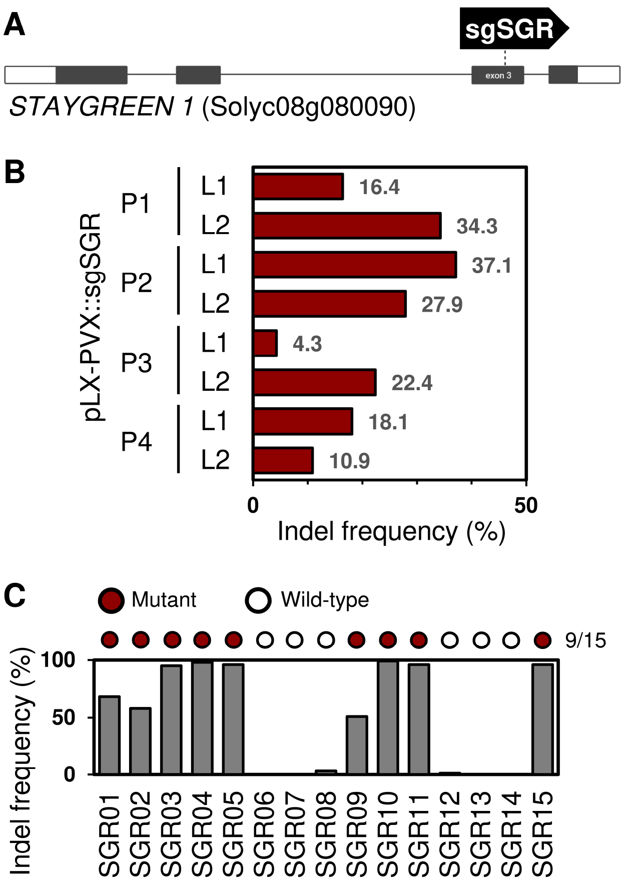

Figure S2. VIGE of tomato *STAYGREEN 1* (*SGR1*). (**A**) Genetic structure of the tomato SGR1 (Solyc08g080090) and the pLX-PVX::sgSGR target region in exon 3. (**B**) VIGE of *SGR1* in tomato somatic cells. MT-Cas9 plants were agroinoculated with pLX-PVX::sgSGR; after 21 days, VIGE was assessed in upper uninoculated leaf samples by PCR of an *SGR1* genomic fragment and Sanger trace deconvolution. Indel frequency percentages are shown for four plants (P1 – P4) and two leaves each (L1 – L2). (**C**) VIGE of *SGR1* in regenerated tomato plants. Indel frequency percentages and genotypic classification of 15 regenerated plants are shown.

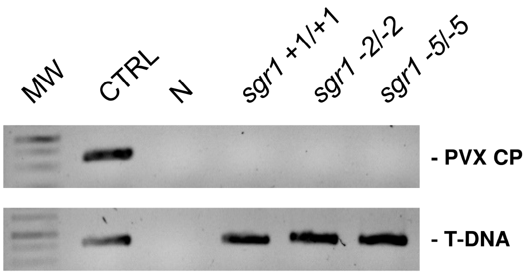

Figure S3. PVX detection in *SGR1* mutant lines. Top, RT–PCR detection of a PVX genomic fragment (CP) from total RNA samples; bottom, PCR detection of a T-DNA fragment from genomic DNA samples; MW, DNA size standards; CTRL, positive control; N, negative control.

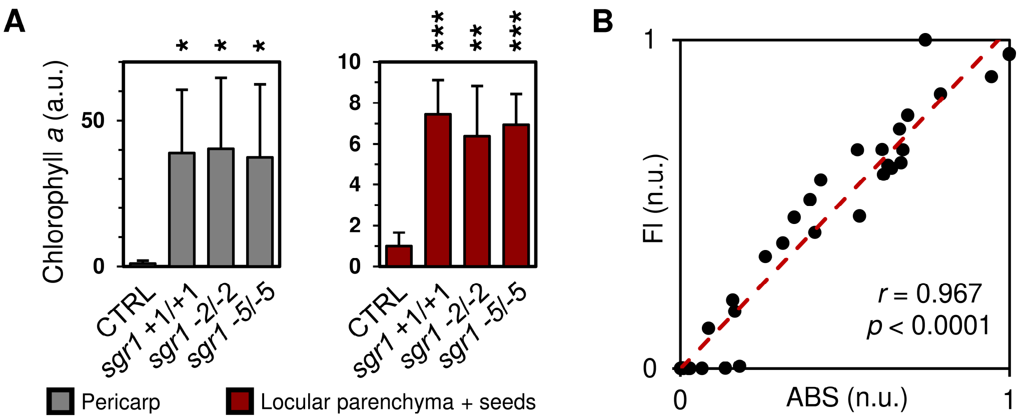

Figure S4. Chlorophyll spectrophotometric quantification in fruits of *SGR1* mutant lines. (**A**) Chlorophyll spectrophotometric quantification. Normalized absorbance-derived amounts of chlorophyll *a* in fruit samples are plotted (mean ± SD, $n = 4$); samples from the unedited MT-Cas9 line were used as the control condition (CTRL); significance levels versus CTRL as per Student's *t*-test are shown; *, $p \leq 0.05$; **, $p \leq 0.01$; ***, $p \leq 0.001$. (**B**) Linear relationship between chlorophyll *a* normalized units (n.u.) determined by spectrophotometric (ABS) and fluorometric (FI) analyses ($n = 32$); Person's *r* and *p* value are indicated.

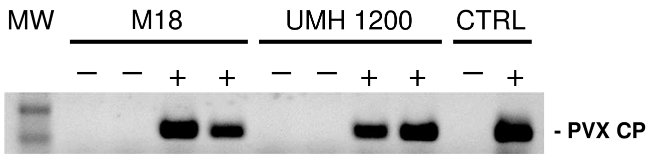

Figure S5. PVX infection of cultivated tomato varieties. PVX was inoculated to plants of the tomato lines Muchamiel 18 (M18) and UMH 1200, an M18 derived line[3] with the introgressed viral resistance *Tm-2*^{2}, *Sw-5*, and *Ty-1* loci. After 3 weeks, total RNA samples were obtained from upper uninoculated leaves and RT–PCR detection of a PVX genomic fragment (CP) is shown. Each lane is from a single plant sample of the untreated (—) or PVX-inoculated (+) condition; MW, DNA size standards; CTRL, samples from control plants.

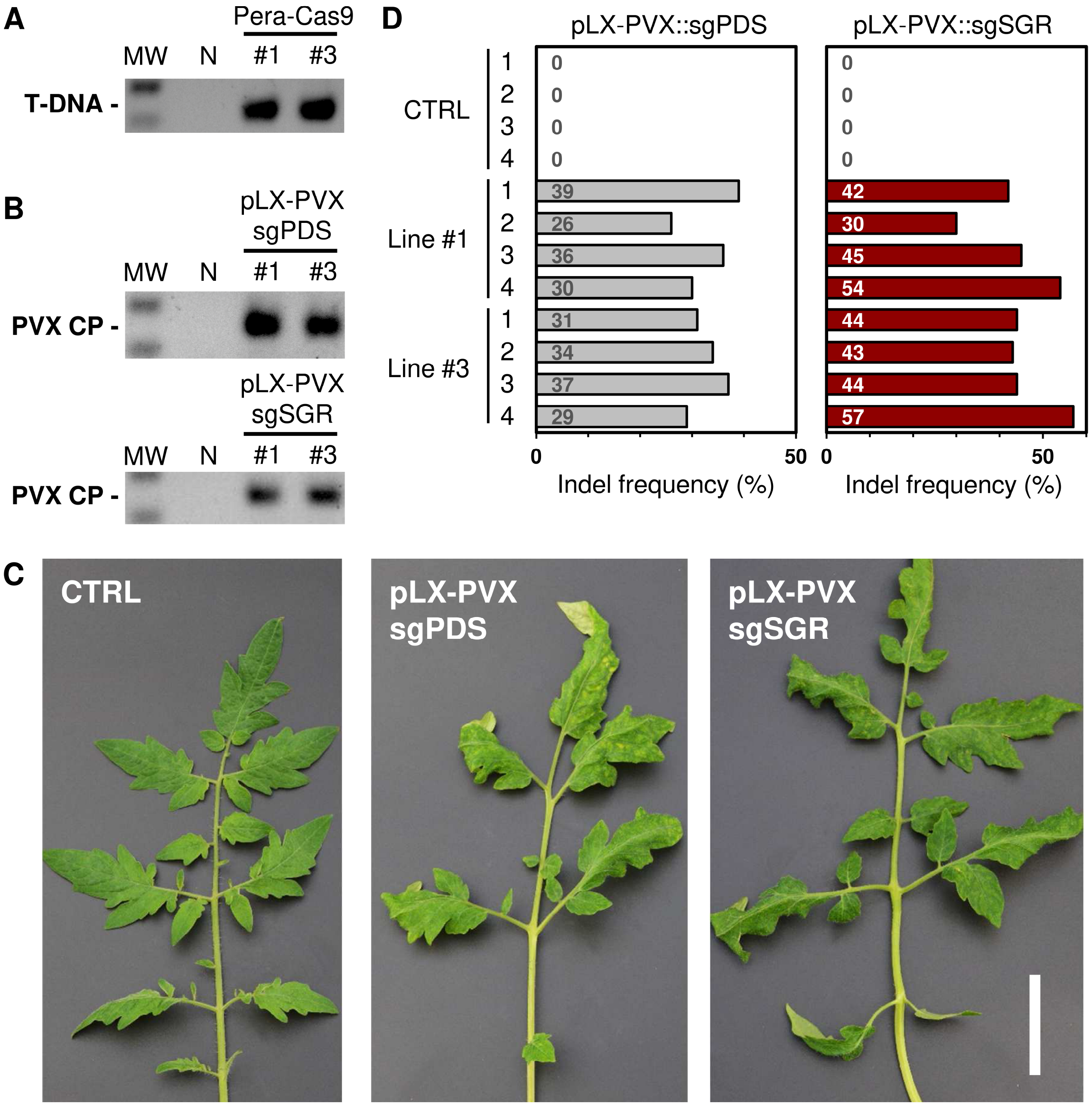

Figure S6. Somatic gene editing of Pera, a cultivated traditional tomato. (**A**) Generation of the transgenic Cas9-expressing lines #1 and #3 of the traditional cultivar Pera (Pera-Cas9). Gel shows PCR detection of a T-DNA fragment from genomic DNA samples; MW, DNA size standards; N, negative control. (**B**-**C**) Agroinoculation of the Pera-Cas9 lines #1 and #3 with pLX-PVX vectors for sgRNA expression. T_1 seedlings were agroinoculated with pLX-PVX::sgPDS and pLX-PVX::sgSGR. After 3 weeks, total RNA samples were obtained from upper uninoculated leaves and RT–PCR detection of a PVX genomic fragment (CP) is shown in **B**; MW, DNA size standards; N, negative control. Phenotype pictures are shown in **C**; scale bar = 5 cm. (**D**) VIGE of *PDS* and *SGR1* in somatic cells of Pera. Three weeks after inoculation, VIGE was assessed in upper uninoculated leaf samples by PCR of *PDS* and *SGR1* genomic fragments and Sanger trace

deconvolution. Indel frequency percentages are shown for the Pera-Cas9 lines #1 and #3, and four plants per condition (1-4); CTRL, uninoculated Pera-Cas9 samples.

SUPPLEMENTARY REFERENCES

1 Pan C, Ye L, Qin L *et al.* CRISPR/Cas9-mediated efficient and heritable targeted mutagenesis in tomato plants in the first and later generations. *Sci Rep* 2016; **6**: 24765.

2 Gianoglio S, Comino C, Moglia A *et al.* In-depth characterization of *greenflesh* tomato mutants obtained by CRISPR/Cas9 editing: a case study with implications for breeding and regulation. *Front Plant Sci* 2022; **13**: 936089.

3 García-Martínez S, Grau A, Alonso A, Rubio F, Valero M, Ruiz JJ. UMH 1200, a breeding line within the Muchamiel tomato type resistant to three viruses. *HortSci* 2011; **46**: 1054–1055.
